# Bilateral and unilateral odor processing and odor perception

**DOI:** 10.1038/s42003-020-0876-6

**Published:** 2020-04-01

**Authors:** Tal Dalal, Nitin Gupta, Rafi Haddad

**Affiliations:** 1 The Gonda Multidisciplinary Brain Research Center, Bar-Ilan University, Ramat-Gan, 5290002 Israel; 2 Department of Biological Sciences and Bioengineering, Indian Institute of Technology Kanpur, Kanpur, Uttar Pradesh 208016 India

**Keywords:** Neural circuits, Olfactory system, Learning and memory

## Abstract

Imagine smelling a novel perfume with only one nostril and then smelling it again with the other nostril. Clearly, you can tell that it is the same perfume both times. This simple experiment demonstrates that odor information is shared across both hemispheres to enable perceptual unity. In many sensory systems, perceptual unity is believed to be mediated by inter-hemispheric connections between iso-functional cortical regions. However, in the olfactory system, the underlying neural mechanisms that enable this coordination are unclear because the two olfactory cortices are not topographically organized and do not seem to have homotypic inter-hemispheric mapping. This review presents recent advances in determining which aspects of odor information are processed unilaterally or bilaterally, and how odor information is shared across the two hemispheres. We argue that understanding the mechanisms of inter-hemispheric coordination can provide valuable insights that are hard to achieve when focusing on one hemisphere alone.

## Abstract (web summary)

Dalal et al. provide a thorough review of bilateral hemispheric interactions in the context of odor perception and memory. They summarize reports on anatomy, physiology and behavior related to olfaction. This review provides insights on how odor information can be processed unilaterally and/or bilaterally despite the lack of topographical organization.

## Introduction: inter-hemispheric communication and perceptual unity

In the early 1940s, patients suffering from severe untreatable epileptic seizures underwent a pioneering operation in which their corpus callosum was severed. While this mitigated the epilepsy syndromes, it generated strange phenomena: each hemisphere seemed to have its own separate perception, concepts, and even actions. When such split-brain patients were shown an image of an object in their left visual field, they could not vocally name what they had seen. This is because information about the image seen in the left visual field is sent only to the right side of the brain, while the speech-control center is usually on the left side of the brain. Nonetheless, patients could pick up the correct object with their left hand because it is controlled by the right hemisphere^1^. Some of the surgery-induced deficits improved over time, but they illustrate one important principle: the fibers that interconnect the hemispheres are responsible for transferring information from one side to the other. These connecting fibers may be required to achieve perceptual unity, at least in some sensory modalities.

But how is perceptual unity achieved? There are several possible ways to achieve perceptual unity in the healthy brain. One way is to ensure that sensory information is transmitted to both hemispheres directly from the sensory organs. This is how auditory information is unified across hemispheres: information from both ears converges to the left and the right Superior Olivary Complexes^2^ such that signals sent to the auditory cortices contain integrated information from both ears. However, not all sensory information is projected bilaterally. For example, ganglion cells activated by the left visual field project to the right hemisphere only and vice versa^3^. By contrast tactile and noxious sensory information is projected unilaterally^4–6^. To achieve perceptual unity in these sensory modalities, downstream neurons must be interconnected across hemispheres. This is accomplished by axonal connections between the two sides of the nervous system, known as commissures.

Commissural systems are present throughout vertebrate and invertebrate species. The commissure fibers include the corpus callosum, the anterior and posterior commissures and the hippocampal commissure. The corpus callosum, which is exclusive to eutherian (placental) mammals, is the largest commissural track. The anterior commissure (AC) connects the temporal lobes, in which the olfactory cortex resides. There are also commissure tracks that interconnect subcortical regions.

In general, commissural systems connect homotypic regions in each hemisphere^7,8^. Hence, information sharing between hemispheres is greatly simplified when the sensory information is topographically organized in the cortex according to some physical property, as is the case for almost all sensory systems^9^. However, there is no known topographical organization of odor space in the main olfactory cortices (OCs)^10–15^. This raises the intriguing question of how odor information is shared between the two olfactory cortices.

Studying how sensory information is shared through interconnected cortical regions provides a unique window into the processes involved in learning and memory, information coding, and perception. For example, learning-related plastic changes could occur in upstream neurons, before the sensory information is shared between the hemispheres. This would result in unilateral memories, whose analysis could shed light on how memories are encoded, where they are formed and how they are accessed. Consider another example: When conflicting stimuli are provided to the left and the right hemispheres, a perceptual conflict sometimes occurs known as binocular rivalry or binaural rivalry, in which only one of the two stimuli is perceived at a time^16,17^. This phenomenon can be used to reveal which hemisphere is more dominant and how dominance is represented and achieved.

Here we review recent advances in research on the unilateral and bilateral processing of odor information and their implications for learning, memory and perception. We start with a short review of the olfactory system with a focus on the bilateral connections. We then discuss what can be learned from delivering odors either unilaterally or bilaterally. We then discuss results from experiments on rodents that are beginning to reveal the underlying neural circuits.

## An overview of the olfactory system

In mammals, smelling begins with the inhalation of odorized air into the nasal cavity. Inhalation is followed by exhalation to form a respiration cycle. The typical awake freely breathing respiration rates are ~0.1–0.3 Hz in humans and ~1–3 Hz in mice^18–20^. The flow of air in one nostril is greater than the other because there is a slight turbinate swelling in one nostril that switches between the nostrils every few hours^21–25^. The nasal cavity is a complex organ containing several passages and turbinates. In the upper part of the nasal cavity, a dense sheet of neurons forms the olfactory epithelium. The epithelium contains millions of olfactory sensory neurons (OSNs), each expressing just one olfactory receptor (OR) out of a large repertoire estimated to include ~1000 different ORs in mice^26^. The left and the right olfactory epithelia are separated by a septum that prevents direct communication between them. The interaction between an odorant molecule and an OR triggers a cascade of events that transduces this interaction into an equivalent neural signal^27^. An OR can respond to several odorants. The set of all such odorants can be considered the OR’s receptive field. Each OSN sends out only one unbranched axon unilaterally to a structure called the glomerulus, which is located on the olfactory bulb’s (OB) surface^28^. Glomeruli are spheroid or ellipsoid conglomerates of neuropil in which synapses are formed between the OSNs’ axons and OB neurons. OSNs projecting to one glomerulus all express the same OR^29,30^. Thus, each odor can be uniquely represented by the set of activated glomeruli, which in turn represents the set of ORs to which that odorant binds. The time of glomeruli activation is believed to be part of the odor code^20,31–34^ (and see ref. ^35^). Glomeruli in the left and right OBs that receive input from the same OR type are located in mirror-symmetric locations conserved within the species^36–38^. While each OSN projects to only one glomerulus, OSNs expressing the same OR project to one of two glomeruli; one on the medial side of the bulb and one on the lateral side of the bulb, forming two mirror-symmetric maps^30,39,40^.

Each glomerulus is innervated by a few dozen mitral and tufted (M/T) cells^5^. M/T cells interact with several inhibitory neurons in the OB^41–52^ and carry the processed olfactory information from the OB to several olfactory cortical areas including the anterior olfactory nucleus (AON), piriform cortex (PC), amygdala, entorhinal cortex (Ent), tenia tecta (TT), and the olfactory tubercle (OT)^53^. The AON is located caudally to the OB and rostrally to the PC, and is therefore the most anterior region receiving direct information from the OB. The AON is divided into four main regions corresponding to their positions (pars dorsalis, medialis, lateralis and ventralis; together termed pars principalis) and another external region called pars externa (AONpE). The AONpE is a band of neurons circumscribing the lateral and dorsal parts of the anterior AON. Early anatomical studies showed that the projections of M/T cells to the AONpE maintain a rough topography of the glomerulus positions in the lateral-medial and ventral-dorsal axes^54^. A more recent study showed that it also preserves the anterior–posterior axis^55,56^

The PC is the most highly investigated olfactory cortex and is believed to serve as an association cortex^57^. The PC is divided into anterior and posterior portions (aPC and pPC). The boundary between the two subdivisions is marked by a dramatic decrease in the thickness of the lateral olfactory tract^58^. The aPC receives more afferent inputs from the OB, whereas the pPC receives mainly associational inputs^59^. With the exception of the AONpE, OC neurons are innervated by several M/T cells^11,12,60,61^. The number of OB inputs that each OC neuron integrates is estimated to range between 4 and 100 and is likely to differ between OC regions^11,60^ (Fig. 2a). An OC neuron typically fires only if several of these inputs are active^60–62^. The exact number of M/T cell connections to a single OC cell, the fraction of these connections that must be active to drive an OC cell, and the different computations employed by different OC cells on these inputs are still open questions.

While the left and right olfactory systems exhibit anatomical symmetry, there are also some noteworthy differences. Several studies in humans have shown that odor recognition involves the right piriform and orbital frontal cortices, while pleasantness rating is predominated in the left PC^63^. Odor discrimination is better when using the left nostril^64,65^. Recent studies in rodents have also revealed lateralization in performing odor discrimination^66,67^ and valence^68^. For a comprehensive review of olfactory lateralization see ref. ^69^.

## Anatomy of bilateral connectivity

**Fig. 1:**
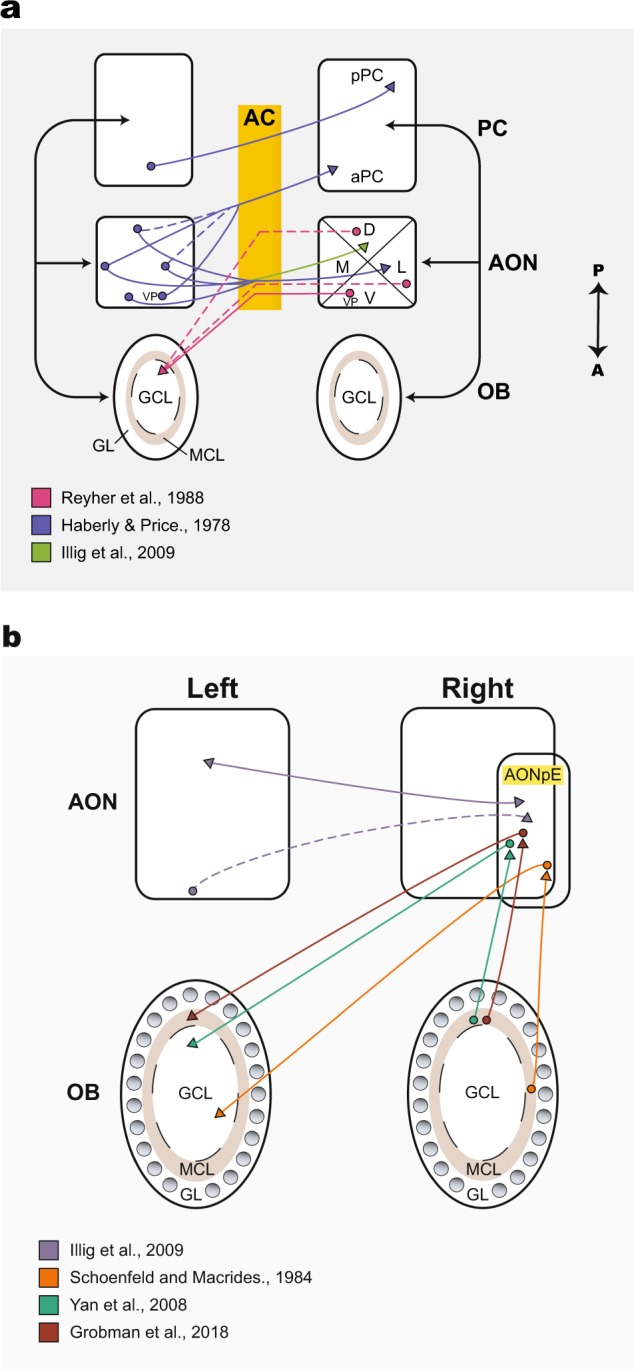
Summary of inter-hemispheric connections in the olfactory system. **a** Schematic representation of the inter-hemispheric connections in the olfactory cortex. The unilateral projections are not shown. Circles represent cell bodies, arrow-heads show the projection target. Solid lines represent dense projections and dashed lines represent minor or weak projections. The green arrow^70^ originates from all subdivisions (D,V,L,M) of the ipsi-AON, and targets the dorsal contralateral AON. Black arrows mark the information flow from the OB to the OC and vice versa. Yellow box represents the anterior commissure (AC). aPC, anterior piriform cortex; pPC, posterior piriform cortex; AON, anterior olfactory nucleus; OB, olfactory bulb; GL, glomerular layer; GCL, granule cell layer; MCL, mitral cell layer;VP, AON pars ventroposterior; D, dorsal; V, ventral; M, medial; L, lateral; A, anterior; P, posterior. **b** Inset of (A), focusing on the interbulbar connections and the bilateral connections between the AON, AONpE and OB. The AONpE preserves the glomerulus topography and projects topographically to the contralateral OB beneath the iso-functional glomeruli. Double-headed arrow denotes reciprocal connections between the AONpE and the AON pars dorsalis. AONpE, AON pars externa.

The bilateral connectivity between the two olfactory cortices is mediated by the anterior commissure (AC). Most bilateral connections are mediated by the AON which has extensive projections to the contralateral AON, PC, and OB (Fig. 1). Anatomical studies utilizing anterograde and retrograde tracing to investigate the projections to the contralateral hemisphere have found that the AON pars dorsalis and pars lateralis receive dense projections from every subregion of the contralateral AON including the AONpE^70,71^ (Fig. 1a–b). The AON constitutes the largest source of centrifugal inputs to the OB^72–74^ (Fig. 1). The pars dorsalis & lateralis were shown to project bilaterally to the OBs but asymmetrically (ipsilateral projections are more dense), whereas projections to the OB from the posterior part of the pars ventralis (referred to as the pars ventroposterior) are bilateral and symmetric^75–78^. The pars ventroposterior together with pars lateralis, dorsalis & medialis project to the contralateral aPC^79,80^ (Fig. 1a).

In addition, the AONpE receives large inputs from the contralateral pars dorsalis and additional minor inputs from the contralateral pars ventralis^70^ (Fig. 1b). The AONpE projects topographically to the contralateral granule cell layer (GCL) underneath the iso-functional glomeruli^54,56,81–83^ (Fig. 1b).

PC neurons are also interconnected. Anterograde injections to the aPC on one side terminate in the contralateral pPC. Retrograde injection to the contralateral pPC revealed that the input originates in the ipsilateral aPC in layer IIb^71^. In addition, aPC receives input from the contralateral nucleus of the lateral olfactory tract^80^. According to Haberly and Price, the TT and the OT are not involved in inter-hemispheric communication^71^. All the currently known inter-hemispheric connections between the OBs, AONs and PCs are summarized in Fig. 1. To summarize, there are many commissural connections in the olfactory cortex. These intricate bilateral connections are likely to play a central role in the exchange of olfactory information and the formation of a unified odor percept.

## Functional bilateral connectivity

The ways in which the contralateral projections affect odor responses has been much less investigated. One study showed that AON back-projections to the contra-OB is coupled to inhalation, and is higher in the awake state than in the anesthetized state^76^. AON-to-contra-OB inhibitory and excitatory back-projections were shown to be odor dependent^76^. Optogenetic activation of AON back-projections to the contra-OB have been found to elicit inhibition in M/T cells and excite local inhibitory neurons^75^. A more recent study showed that optogenetic activation of AONpE back-projections to the contra-OB can also excite M/T cells^81^.

**Fig. 2:**
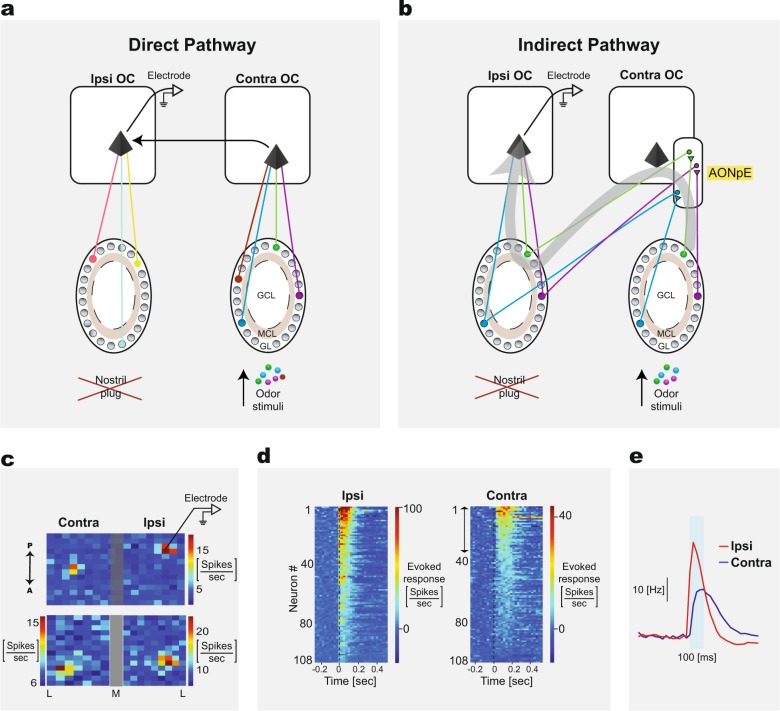
The direct and the indirect pathways underlying functional bilateral connectivity. **a** OC neurons receive inputs from several ipsilateral M/T cells and different OC neurons integrate from a varied number of different M/T cells. OC neurons can receive input directly from the contralateral OC neurons (black arrow). The direct pathway: An odorant inhaled through the contra-nostril activates a glomerulus pattern in the contra-bulb (four colored circles in the glomerulus layer). This pattern then activates several M/T cells in the contra-OB which converge to a single contra-OC neuron (black pyramid). This cortical neuron in turn projects to a corresponding ipsilateral OC neuron via the AC. The big X represents an occluded nostril. **b** Summary of the indirect pathway. Odorants inhaled through the contra-nostril activate a glomerular pattern in the contra-OB. This pattern activates several M/T cells in the contra-OB, which results in activating mirror-symmetric M/T cells in the ipsi-OB by first activating the contra-AONpE neurons. These mirror-symmetric M/T cells in the ipsi-OB converge to an ipsi-OC neuron (black pyramid), leading to sharing the odor code across the two hemispheres. In addition, the odorant-activated contra-M/T cells converge to a contra-OC neuron (not shown). Thick gray arrow depicts the course of the indirect pathway. **c** Two examples of ipsi- and contralateral light activation maps of M/T cells. Each pixel represents the average firing rate caused by ~50 optogenetic stimulations of each spot on the grid. Mice expressing ChR2 in M/T cells were used. The values were obtained using a 200-ms window after light onset. The gray bar marks the estimated boundary between the ipsi- and contralateral bulbs. The example in the upper panel was constructed by scanning both bulbs in the same experiment and the lower panel example was constructed from two independent light-scan experiments of the ipsi- and contralateral bulbs. Panels C-E were taken from^81^. **d** Raster plots of all 108 recorded neurons’ ipsi- and contralateral hotspot responses. Each bin is the average firing ring rate in a 25 ms window. In four neurons the responses to ipsi-light stimulation exceeded 100 spikes/s and were truncated to 100 for better visibility of low firing rates. Double-headed arrow marks the neurons that received significant excitatory input from contralateral light stimulations. **e** The median response for each 25 ms bin of all ipsilateral and contralateral significantly responding neurons. The duration of the stimuli is indicated by the cyan bar. Responses to stimulation from the contra-bulb tended to have a lower peak and lasted longer.

As mentioned above, each OC neuron receives direct inputs from multiple ipsilateral M/T cells and responds only if several of these neurons are active. However, an OC neuron can also respond to an odor delivered to the contra-nostril^84,85^. There are at least two possible pathways by which OC neurons can respond to a contra-nostril odor stimulus. In the direct pathway, an OC neuron receives input from the contra-OC via the AC (Fig. 2a). OC neurons can also receive olfactory input indirectly. A recent study found that optogenetically activated contra-M/T neurons can activate mirror-symmetric ipsi-M/T neurons via the contra-AONpE^81^. About one-third of the recorded M/T cells were activated by optogenetically activating contra-M/T cells and these activations were relatively weaker than ipsi-activated M/T cells (Fig. 2c–e). Connected M/T cells responded to similar odors, although odor delivered to the contra-nostril typically elicited weaker responses. A recent study found similar bilateral symmetric excitatory link between zebrfish M/T cells^86^. An OC neuron can thus be activated by a contra-odor indirectly through the contralateral M/T cells that responded to the odor and then activate the corresponding ipsilateral M/T cells via the AONpE. The indirect pathway is summarized in Fig. 2b.

The direct pathway predicts that an OC neuron will have different ipsilateral and contralateral odor receptive fields. This is because OC neurons receive their input from a random set of M/T cells. Thus, an ipsi-OC and a contra-OC neurons are likely to receive their inputs from a different set of glomeruli (Fig. 2a). The indirect pathway predicts that an OC neuron will have similar odor receptive fields for ipsilateral and contralateral odors because the neuron is either directly activated by the ipsi-M/T cells it is connected to, or indirectly through the symmetric set of contra-M/T cells that also activate the mirror-symmetric ipsi-M/T cells. Since only approximately one-third of the M/T cells are connected by the indirect pathway to the contra-M/T cells, and those that are connected have relatively weak connections^81^, the indirect pathway also predicts that only a subset of OC neurons will respond bilaterally and that these responses will be weaker than ipsi-odor responses (Fig. 2b). A study that examined the response of AON pars principalis neurons to a large set of odors found that 60% of the neurons responded to ipsilateral and contralateral odors^85^. Responses to contra-odors were generally weaker. This result is inline with the indirect pathway. However, a very recent study found that a substential number of AON and aPC neurons responded to ipsi- and contralateral odors and that these responses are of similar magnitute^87^. This similarity in bilateral odor receptive fields suggests that AON and aPC neurons receive contra-input through the direct pathway. Another study found that some aPC neurons responded only to contra-odor or only to ipsi-odor, but a few responded only when the odor was delivered bilaterally^84^. This finding might suggest that some aPC neurons receive direct input from the contra-OC. However, since only one odor was used in this study, a comprehensive comparison of odor receptive fields could not be made. Future experiments that will examine the ipsi- and contra- odor receptive fields of neurons located in different OC regions to a battery of odor stimuli delivered at different concentrations to each nostril will shed new light on the bilateral functional connectivity in the olfactory system and the role of the indirect and the direct pathways.

## How is perceptual unity achieved in olfaction?

**Fig. 3:**
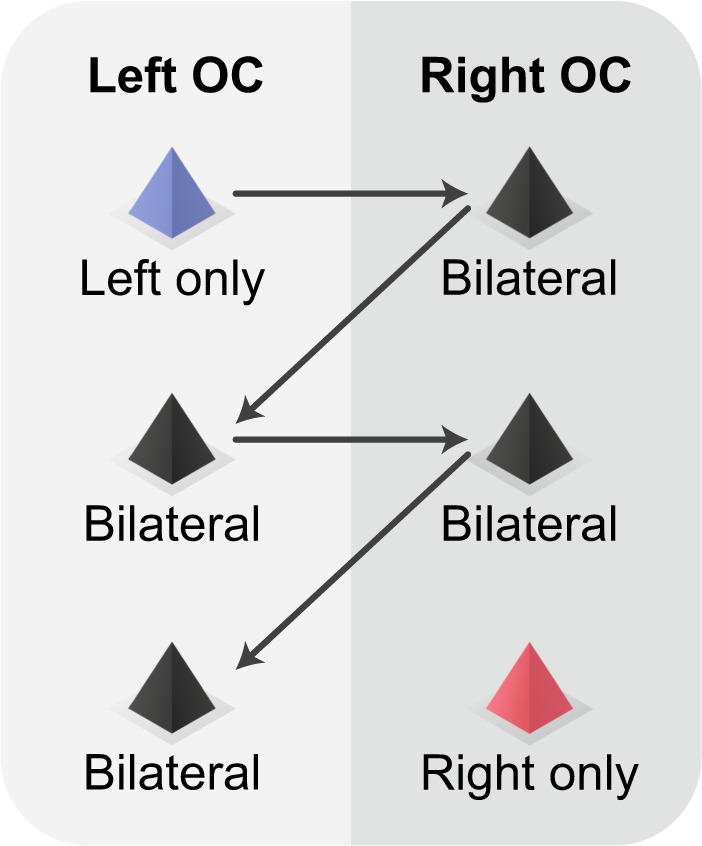
Inter-OC connections may enable sharing of odor representation across the two hemispheres. An odorant inhaled through the left nostril activates several left-OC neurons. These neurons in turn activate several right-OC neurons through non-reciprocal connections, but lead to an odor representation in the right-OC that is similar to the odor representation when the odorant is delivered directly through the right nostril. Black pyramids represent neurons activated regardless of whether the odorant was delivered to the left or the right nostril. Blue and red pyramids represent neurons activated by left-only or right-only delivered odorants, respectively.

The indirect pathway suggests a simple solution to the perceptual unity problem in olfaction. Assuming that an odor inhaled through one nostril is encoded by a small set of strongly activated glomeruli ipsilateral to that nostril, the M/T cells innervating these glomeruli will activate their mirror-symmetric contra-M/T cells, which will result in sharing the odor code across both bulbs (Fig. 2b–e). OC neurons in each hemisphere can then read out the same glomerular pattern from their corresponding OB. These neurons will be activated regardless of the odor entry via the left, the right, or both nostrils. Consistent with this model, it was found that the neural response elicited by contra-odors is sufficient to decode the odor identity by a classifier trained on the neural responses elicited by ipsi-odors^81^. Thus, the indirect pathway is sufficient to share odor identity across bulbs. This circuit enables sharing of odor information across hemispheres in the absence of a cortical topographical organization and homotypic projections, suggesting that olfactory glomerular maps are the equivalent of cortical sensory maps found in other senses. Thus, although there is no known topographical organization of OC neurons, the glomerular organization in the OB together with the indirect pathway can maintain the continuity of sensory maps across hemispheres as in other sensory systems. Alternatively, it is possible that odors are shared across hemispheres via the direct pathway. In this scheme, the AC must preferably interconnect neurons that have similar odor receptive fields despite the lack of topographical organization (Fig. 2a). This can be achieved in two steps. The first would form bilateral connections in an activity-independent manner to produce a rough map characterized by excessive inter-hemispheric connections. The second would involve activity-dependent pruning leaving behind mostly bilateral connections of similar receptive fields. It may be possible to have a similar set of neurons activated by ipsi- and contra-odors, even if the activity-dependent inter-OC connections are not reciprocal (Fig. 3). The exisitance of extensive bilateral connections suggests that the direct pathway plays an important role in bilateral information transfer^87^.

Random connectivity between OB and OC neurons^10–12^ makes the task of achieving perceptual unity more difficult. One way of overcoming this randomness in connections has been suggested in recent modeling studies^88,89^. The idea is that two neurons, each located downstream of PC neurons in a different hemisphere, can have similar odor receptive fields, despite the variability in the OB–OC connections in the two hemispheres, if they integrate odor information from a large number of OC neurons. This intriguing idea can explain how the mushroom body output neurons located in the two hemispheres of the *Drosophila* brain can have similar receptive fields^90^. Furthermore, it provides an explanation for how similar odor responses, and therefore olfactory behaviors, can be achieved across subjects. However, it does not explain how a unilateral odor memory is accessed bilaterally (see below and ref. ^91^).

## How inter-nostril interactions affect odor perception

Since the brain must achieve perceptual unity for proper functioning, delivering different odors to the left and right nostrils and evaluating the resulting perception can reveal important sensory processing principles. A study examined the perceived odor intensity in humans when the same odor was delivered at different concentrations to the two nostrils, and found that the intensity perceived when inhaling the odor with both nostrils open was slightly lower than the sum of the intensities perceived when inhaled with the left nostril and the right nostril individually^92^. This result may suggest that the perceived odor intensity reflects the overall neural activity in both hemispheres summated sub-linearly or that there is some weak inter-hemispheric suppression that reduces the overall perceived intensity.

Another study examined how odors are perceived when each nostril receives a different odor^16^. The experimenters delivered Phenyl Ethyl Alcohol (PEA, a rose-like smell) to one nostril and n-butanol (a marker like -smell) to the other. Subjects were asked to report if the odor smelled was more like a “rose” or “marker”. They found that the subjects perceived either the marker or the rose odor^16^. The odor perception was constant within a sniff and could change between sniffs. This suggests that although each hemisphere presumably perceived a different odor, only one percept dominated rather than both contributing equally. Recording the neural activity of OC neurons during unilateral and bilateral stimulations could shed light on the possible mechanisms underlying this binaral rivalry phenomenon and the neural substrate of perception. One possibility is that there is contra-hemispheric inhibition among PC neurons resulting in a winner-takes-all arrangement, such that the left-odor dominates if the left-odor responding neurons inhibit the right-odor responding neurons, and vice versa.

## Cross-nostril odor adaptation

In all sensory systems, prolonged exposure to a stimulus causes adaption such that the detection threshold of the adapted stimulus is increased. Olfaction is no exception: prolonged exposure to an odor increases the detection threshold to that odor^93^. Adaptation to one odorant does not substantially affect the detection threshold of another structurally dissimilar odorant^93–95^. Neural adaptation have been shown to occur at multiple levels from OSN to M/T and PC neurons^96–101^. Adaptation of aPC neurons seems to be odor specific^98,99^. This finding suggests that the adaptation is not a result of a global reduction in neuronal excitability or an increase in phasic recurrent inhibition. In vitro and in vivo experiments showed that the reduced response of aPC neurons during adaptation is due to synaptic depression of cortical afferents^102^. Thus, adaptation to one odor will cause adaptation to another odor only if they activate a similar set of M/T cells. Investigating cross-nostril adaptation can provide additional insights as to where adaptation occurs, the underlying mechanisms, and to what extent odor information is shared across hemispheres. Studies that investigated cross-nostril adaptation in humans found that an odorant stimulus presented to one nostril caused bilateral adaptation, although adaptation was stronger and longer in the ipsilateral nostril^16,92^. 12-day-old rat pups that were habituated to an odorant on one side were not habituated to the odorant on the other side when the AC was transected before habituation started^103^. This finding implies that the adaptation is transferred to the other side through the AC. If ipsilateral odor adaptation is a result of ipsi-M/T afferent synaptic depression, how does cross-nostril adaptation occur? One possible mechanism is that contra-M/T cells that were activated via the indirect pathway also undergo synaptic depression. Since only a subset of contra-M/T cells are active during ipsi-nostril odor stimulation^81^, cross-nostril adaptation will be weaker than ipsi-nostril adaptation. Alternatively, adaptation could be the result of reduced excitability caused by the direct pathway. An experiment in which contra-M/T neurons are silenced during ipsi-nostril adaptation could determine the exact mechanism.

## Cross-nostril odor detection

While prolonged continuous exposure to an odorant may increase its detection threshold, short and repeated brief exposures to an odorant over days may have the opposite effect, enabling detection of odorants that were not detected previously^94,104,105^. About 30% of the adult population cannot smell the molecule androstenone but can be trained to detect it^106–108^. The mechanism underlying this phenomenon is unknown. One study found that people with specific anosmia to androstenone who learned to detect androstenone with one nostril plugged could detect it with their previously plugged nostril^108^. This may suggest that learning to detect androstenone occurs at a central location that has bilateral access to odorant stimuli or that the changes are shared bilaterally via the indirect pathway. Alternatively, it is possible that over the training days the number of OSNs expressing the ORs that are sensitive to androstenone in both epithelia slowly increased following some bilateral upregulation process. Consistent with this mechanism, changes in the activity of OSNs in the epithelium of mice trained to detect androstenone were reported using electro-olfactography^107^. Measuring changes in the activity of OSNs and M/T cells in the plugged nostril may demonstrate that these changes occur bilaterally.

## Ipsilateral and contralateral odorant discrimination and generalization

As in the case of odorant detection thresholds, odor discrimination can also be improved with training^109,110^. Enantiomers are molecules that are mirror-reflections of each other. There are many enantiomers in nature. Some enantiomers elicit different smells and others elicit smells that are indistinguishable to humans^111^. However, people can be trained to discriminate between two previously undistinguishable enantiomers^112,113^. Subjects that were trained to discriminate between two previously undistinguishable enantiomers by exposing them to the odorants repeatedly over ~12 days with one nostril closed, failed to discriminate between the enantiomers with the previously closed nostril^112^. This finding suggests that changes occurring during the learning process were mostly unilateral. One possible mechanism to explain this result is that learning to discriminate the enantiomers involves changes in the interactions between M/T and local inhibitory neurons in the ipsi-OB which enhance the differences in the representations of the two previously indistinguishable odorants. Evidence of the involvement of local inhibitory neurons in learning to discriminate highly similar odorants has been reported^114–117^. These changes in local interneurons might only occur on the ipsi-side (perhaps because they might need high activation). Bilateral recording of M/T and OC cell responses in mice that learn to discriminate enantiomers with one nostril plugged could shed light on the exact locus of this learning process and its underlying mechanism.

It also remains unclear whether stimulus generalization is also nostril-specific. Generalization is the process of manifesting a conditioned response to a stimulus that is similar to a previously conditioned stimulus^118–120^. For example, pairing an odorant with a mild electrical shock results in freezing behavior to that odorant and similar odorants^120^. Generalization is a key process for survival. Determining whether generalization is nostril specific can further help reveal the mechanism underlying it.

## Unilateral and bilateral odor memories

Adaptation is a simple form of memory that can be explained by synaptic depression. Learning to detect a previously undetected odorant or learning to discriminate between two very similar odorants requires mechanisms that involve changes in presumably central and peripheral circuits. Associative memory—learning to remember the relationship between two unrelated events—is an even higher level of memory and is likely to involve multiple brain regions. Can associative memory be confined to one hemisphere of an intact animal?

**Fig. 4:**
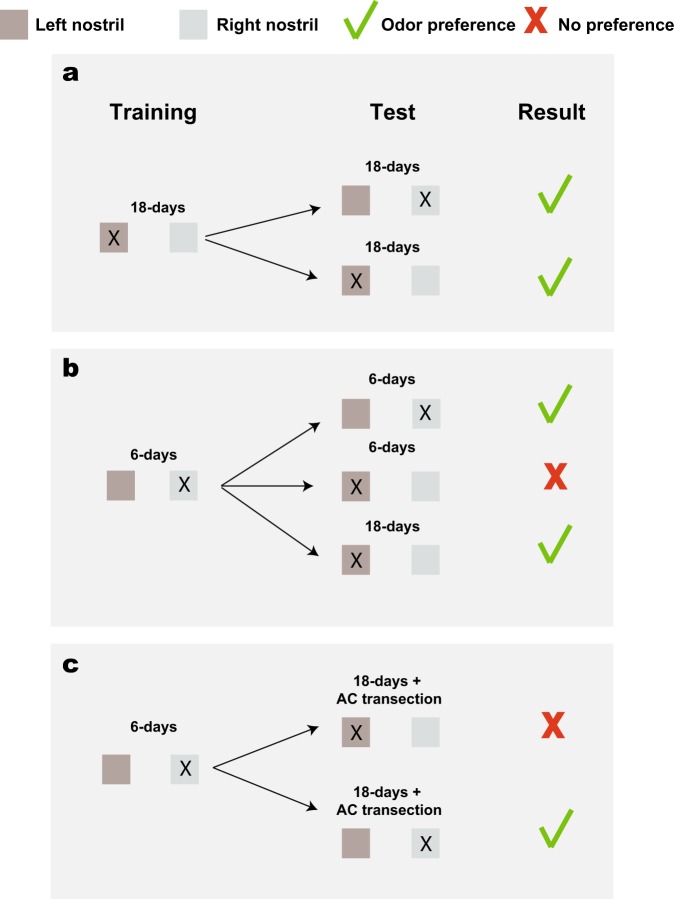
Unilateral and bilateral memory accesses. **a** Rat pups were conditioned to a cedar odorant by pairing it with a milk reward. 18-day old pups that were trained with one nostril plugged (marked with a black X) showed a preference for the cedar odorant with either the trained or the untrained nostril. Green V, preference to cedar odorant; Red X, no preference. **b** 6-day old pups did not show preference for cedar with the untrained nostril but did show preference when reaching the age of 18 days. This suggests that a unilateral odor memory is either bilaterally accessible at the age of 18 days or that it was transmitted to the contralateral side. **c** 6-day old pups trained with one nostril plugged could not retrieve the memory at 18 days if the AC is cut at that age. Thus, the memory is stored unilaterally and is not transmitted to the contralateral side although it can be accessed through the AC from the contralateral side.

Kucharski and Hall investigated whether odorant association memory can be formed unilaterally. Their main training paradigm was to associate a milk reward with cedar odorant in rat pups. This was achieved by pairing the cedar odorant with intraoral infusions of milk while one nostril was occluded and then testing this odorant association by measuring the pups’ orientation towards cedar. 18-day-old pups trained with one naris closed showed increased preference for cedar odorant with both the trained and the untrained naris (Fig. 4a)^121^. This is expected, as odorant smelled with one nostril should be shared with the other hemisphere to enable perceptual unity. 6-day-old pups showed increased preference to cedar odorant only when tested with the trained naris but not with the untrained naris at the same age (Fig. 4b)^122^. This is also expected: as the AC is fully developed only after the age of 12 days^123^, 6-day-old pups are virtually split-brain rats with regard to their olfactory system and therefore odorant preference was not transmitted from one hemisphere to the other. Strikingly, these 6-day-old pups showed associative memory for the cedar odorant with the untrained naris when they reached the age of 18 days (Fig. 4b)^91,121^. This finding implies that the unilateral odorant association memory either became accessible from the contra-side or that it was transferred somehow to the contra-side. To determine which was correct, the authors dissected the AC of 18-day-old pups after they had learned the association for the cedar odorant with one nostril closed at the age of 6 days (Fig. 4c). The rats failed to show associative memory for the odorant with the untrained nostril although they continued to show the memory with the trained nostril. Hence, at least in this training paradigm, unilateral training forms a unilateral odor memory but this memory can be accessed from the untrained side through the AC. A more recent study showed that bilateral lesion of the AONpEs had the same effect as sectioning the AC^56^. A mouse placed in a maze with two arms received a mild electric shock following the presentation of odorant A or B in one of the arms. The shock was delivered in the same arm as the odorant when odorant A was delivered, and in the opposite arm when odorant B was delivered. The mice quickly learned to run to the other arm following the presentation of odorant A and to stay in the arm that they were in when odorant B was delivered. However, mice in which both AONpEs were lesioned *before* training (with one nostril) did not transfer this learning to the other hemisphere^56^. This finding suggests that AONpE is required for contralateral memory access or transfer. Since AONpE mainly projects topographically to the contralateral OB^56,70,74,81^ it is reasonable to assume that the indirect pathway is used for this memory retrieval. In contrast, one study found that mice could access a unilateral odorant association memory even after the olfactory peduncle was transected in the trained side^124^, which should block access to the trained side OB. This result implies that odorant association memory can be accessed without accessing the trained OB, lending weight to the direct pathway hypothesis for memory retrieval. It is possible that both pathways are involved in sharing odorant information across the hemispheres or that different memory types (e.g. fear conditioning versus odorant association) are accessed by different pathways.

## Bilateral processing enables odorant localization

Although the distance between the two nostrils in most mammals is relatively small compared to the distance between the eyes or the ears, the existence of two symmetrical nostrils suggests that animals might use bilateral sampling to extract information about the location of an odorant source. Bilateral odorant sampling is used for odorant localization in many species. For example, *Drosophila* flies whose two antennae were exposed to air streams of different odorant concentrations turned toward the higher concentration^125^. Thus, flies utilize concentration differences between the two antennae to decide on the direction of the odorant source despite the fact that the OSNs in flies project to both sides of the brain. Flies are still able to distinguish the odorant contrast because the synaptic connections made by the sensory axons are stronger on the side where they receive their input, resulting in activation of the downstream neurons slightly earlier on the side with the higher odorant concentration^126^. Among vertebrates, hammer sharks (whose two nares are separated by several centimeters) have been shown to turn toward the side receiving the first stimulus rather than toward the side receiving the stronger stimulus^127^. This suggests that animals can also use bilateral time differences for odorant localization. Plugging one nostril in rats, or spatially reversing the antennae of a locust (by crossing and fixing the antennae), or switching between the left and right nostril inputs in blind moles or human subjects (by inserting plastic tubes into the nostrils and crossing them) hinders their ability to navigate toward the odorant source^128–131^. All these studies demonstrate that animals compare bilateral inputs to extract information about the odorant direction or location. Note that eliminating or crossing bilateral inputs generally disrupts but does not abolish odorant localization in freely behaving animals^128,132,133^. This suggests that animals can use unilateral odorant sampling strategies such as tracking odorant concentration gradients during movement or tracking wind direction to locate an odorant source. Nevertheless, they excel when both their nostrils are open.

The exact nature of the neural mechanism underlying odorant localization remains to be identified. Mice with AC transected or with their AON ablated failed to detect the direction of a novel odorant puff^134^. This strongly suggests that the AON is involved in comparing bilateral inputs. This is consistent with the observation that the majority of inter-hemispheric olfactory connections involve the AON^74^. One study found that neurons in the ventral part of the AONpE are excited by an odorant delivered to the ipsi-nostril and inhibited by the same odorant delivered to the contra-nostril^135^. Delivering different odorant concentrations to the left and the right nostrils showed that these AONpE neuron responses were correlated to the differences between the left and the right odorant concentrations. This finding suggests that ventral AONpE neurons are suitable candidates for comparing the bilateral inputs.

**Fig. 5:**
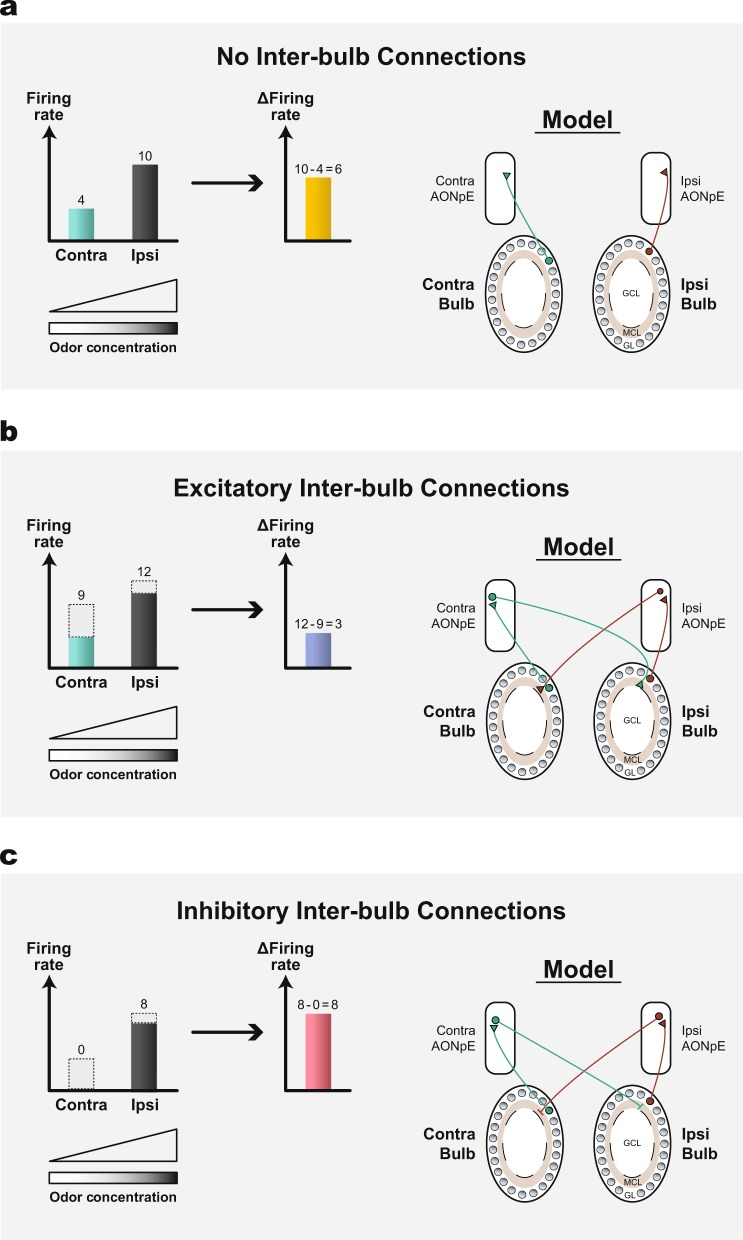
Models of inter-bulb connections and their putative relevance for odorant localization. A high concentration of an odorant is delivered to one side (ipsi) and a lower concentration of the same odorant is delivered to the other (contra) side. **a** Assuming no inter-bulb connections, a higher odorant concentration at the ipsi-nostril leads to higher firing rate of the ipsi-M/T cell than the contra-M/T cell. A large difference enables the animal to localize the odorant on the ipsi-side. **b** Excitatory inter-bulb connections lead to an increase in the M/T cell firing rates on each side, proportional to the excitatory input from the M/T cell on the contra-side via the AONpE. Assuming the ipsi-M/T cell contributes 50% of its firing rate to the contraM/T cell and vice versa, the difference between firing rates will decrease, making odorant localization more difficult. **c** With inhibitory inter-bulb connections, the higher firing rate of the ipsi-M/T cell leads to strong inhibition on the already weakly-active contra-M/T cell, while the ipsi-M/T cell experiences negligible inhibition in return. As a result, the contrast between the two sides is maximized, making odorant localization easier.

The recently discovered excitatory connections between sister-M/T cells across bulbs (the indirect pathway)^81,86^ might be involved in odorant localization (Fig. 2b). However, as discussed in Grobman et al. ^81^ excitatory inter-bulb connections decrease the difference in neural responses between the two sides generated by the difference in odorant concentrations across the two nostrils. This is because each M/T cell increases the response of its contralateral iso-functional M/T cell in a manner proportional to its activity. (Fig. 5a–b; see Grobman et al. for the model details). Inter-bulb inhibition is more likely to facilitate odorant localization since it enhances the difference in neural responses to different odorant concentrations between the two nostrils in a winner-takes-all fashion (Fig. 5c). The abundance of projections from the AONpE to the contra-OB granule cell layer strongly suggests that inter-hemispheric inhibition is involved in this circuit.

## Conclusions

Inter-hemispheric neural interactions serve two major roles. They facilitate perceptual unity in sensory systems in which the sensory organs do not project bilaterally, and enable bilateral comparison of sensory information to extract additional information such as source localization. The neural circuits underlying these somewhat contradictory roles are now starting to be revealed. It is likely that several circuits achieve these goals. Understanding how the two hemispheres work together to achieve these goals is starting to provide valuable insights into long-standing questions in neuroscience: how neurons change during learning, how memories are formed, whether they are stored unilaterally or bilaterally, and how they are accessed. Future studies manipulating the bilateral stimuli or the bilateral information streams, together with bilateral recordings of neural activities in different brain regions, will help answer these fundamental questions.
